# A rare case of the recurrent surgery for cribriform-morular variant of papillary thyroid carcinoma

**DOI:** 10.1016/j.ijscr.2019.11.060

**Published:** 2019-12-06

**Authors:** Keisuke Enomoto, Shunji Tamagawa, Naoko Kumashiro, Kenji Warigaya, Saori Takeda, Mehmet Gunduz, Shin-ichi Murata, Muneki Hotomi

**Affiliations:** a Departments of Otolaryngology-Head and Neck Surgery, Wakayama Medical University, Wakayama, Japan; b Departments of Diagnostic Pathology, Wakayama Medical University, Wakayama, Japan

**Keywords:** CMV-PTC, Recurrence, Total thyroidectomy, Case report

## Abstract

**Introduction:**

The cribriform-morular variant of papillary thyroid carcinoma (CMV-PTC) is an uncommon subtype of PTC, and it is associated with familial adenomatous polyposis (FAP).

**Presentation of case:**

We describe a 32-year-old female who presented recurrent CMV-PTC with FAP. Surgery performed after the recurrent disease resulted in left recurrent laryngeal nerve palsy due to formation of strong scar after initial operation. The histopathological examination revealed the recurrent CMV-PTC without thyroid capsular invasion in remnant thyroid tissue of berry ligament. In addition, the immunohistochemical analysis showed that β-Catenin was diffusely positive in both cytoplasm and nucleus in CMV-PTC tissue.

**Conclusion:**

Total thyroidectomy in CMV-PTC with FAP should be performed at initial surgery due to high recurrence.

## Highlights

• The cribriform-morular variant of papillary thyroid carcinoma (CMV-PTC) is an uncommon variant of PTC, which incidence of CMV-PTC is less than 0.3% of PTC.
• The strong expression of β-Catenin is a hallmark staining in CMV-PTC.
• Total thyroidectomy in CMV-PTC with FAP should be performed at initial surgery due to high recurrence.

## 1 Introduction

The cribriform-morular variant of papillary thyroid carcinoma (CMV-PTC) is an uncommon variant of PTC, and it is associated with familial adenomatous polyposis (FAP), which displays *APC* germ line mutation. CMV-PTC was first described in 1949 as thyroid tumor with another extra colonic manifestation [1], and it belongs to one of the 15 variants of PTC described in the 4th edition of the World Health Organization (WHO) classifications [2]. The relative incidence of CMV-PTC is less than 0.3% of PTC [[3], [4], [5]]. Typically, there is a strong female predisposition (male:female = 1:31) and detected in young adults [6,7]. The prognosis of CMV-PTC is good; low prevalence of lymph node metastasis, low over all recurrence rate, and low disease-related mortality as compared to conventional PTC [6]. We herein report a rare recurrent case of CMV-PTC with FAP. This work has been reported in line with the SCARE criteria [8].

## 2 Presentation of case

A 23-year-old female patient was admitted to our hospital to be followed up after initial near total thyroidectomy diagnosed with poorly differentiated PTC, (pT3, Ex1, pN0) in another hospital. The patient had no complaint with supplementation of levothyroxine sodium hydrate 100 μg/day and alfacalcidol 1.0 μg/day. Because her mother and younger sister had FAP, fiberoptic colonoscopy was planned during postoperative observations of thyroid cancer in our hospital. FAP was diagnosed and gastroenterologist performed endoscopic mucosal resection six times and at 30-years-old, total colectomy was performed.

**Fig. 1:**
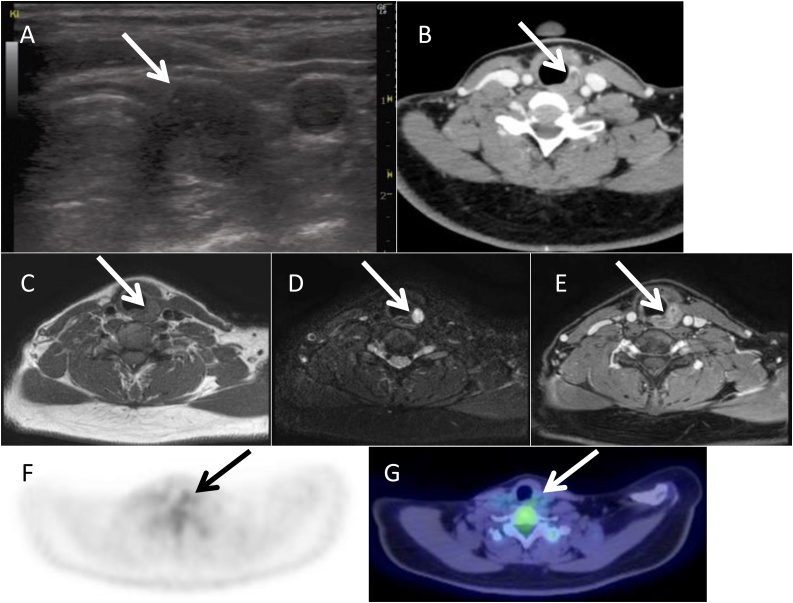
Preoperative image. (A) Ultrasound showed the 15 mm neck mass in the left thyroid bed. (B) A contrast enhanced CT scan of the neck reveal a tumor in the enhanced normal thyroid tissue. (C–E) Axial T1 weighted images demonstrated isosignal intensity mass, which attached to trachea and esophagus. The recurrent tumor was distributed a high signal lesion Axial T2 weighted MR images, and contrast enhanced T1 weighted images clearly showed that tumor did not invade trachea, esophagus, and neck great vessels. (F, G) ^18^F-FDG slightly accumulated to the recurrent tumor site in the positron emission tomography.

At 32-years-old, the 15 mm neck mass was noted in the left thyroid bed during ultrasound examination (Fig. 1A). Blood test was normal including thyroglobulin. A contrast enhanced CT scan of the neck revealed a nodule in the enhanced thyroid tissue (Fig. 1B). MRI image showed that tumor did not show extrathyroidal invasion for trachea, esophagus and neck great vessels (Fig. 1C–E). 2-deoxy-[F-18]fluoro-d-glucose slightly accumulated to the recurrent tumor site in the positron emission tomography (Fig. 1, panels F and G; maximum standard uptake value = 2.52).

**Fig. 2:**
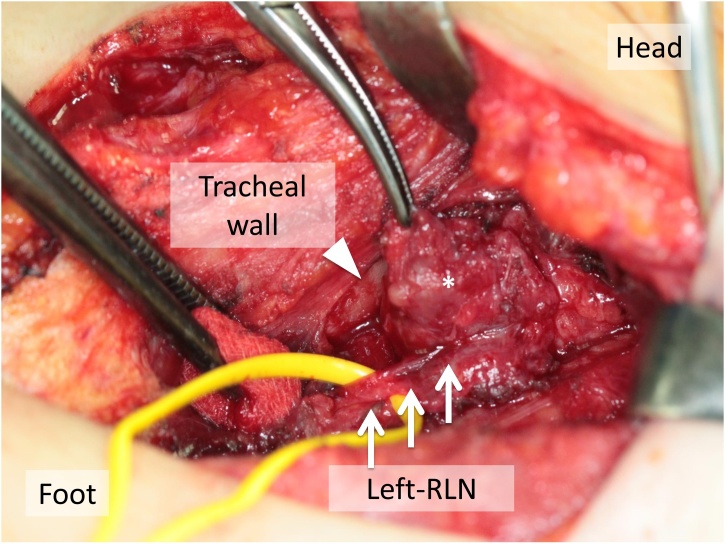
Surgery. The left recurrent laryngeal nerve (RLN; arrows) and tracheal wall (allows head) were strongly attached to remnant thyroid (*).

The fine-needle aspiration cytology showed PTC nuclear features. As the recurrence of CMV-PTC was considered, we performed the re-operation for the remnant thyroid. The left recurrent laryngeal nerve (RLN) was strongly attached to remnant thyroid tissue due to the initial surgery (Fig. 2), and the postoperative RLN palsy was occurred even though using intraoperative neuromonitoring (NIM-3.0 Neuro Monitoring System, Medtronic Xomed; Jacksonville, FL).

**Fig. 3:**
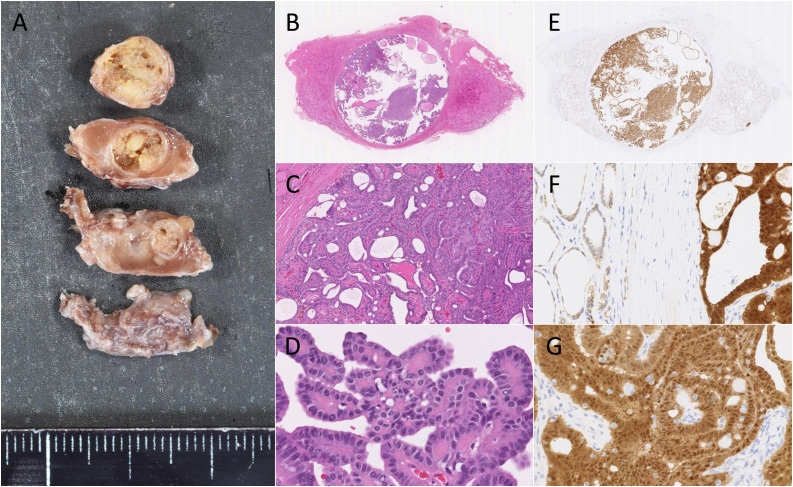
Histopathological analysis. (A) Gross findings. The recurrent CMV-PTC was encapsulated in remnant thyroid tissue of berry ligament. (B–D) Hematoxylin-Eosin staining showed the characteristic cribriform pattern of arrangement of the tumor cells. This is composed cribriform and papillary patterns. (E–G) The immunohistochemical analysis showed that β-Catenin is diffusely positive in both cytoplasm and nucleus of cancer cells in CMV-PTC. (F) However, normal epithelium of thyroid gland shows membranous staining pattern.

A final diagnosis of CMV-PTC was made based on the pathological morphological features, as shown in Fig. 3. The histopathological examination revealed that the recurrent CMV-PTC without thyroid capsular invasion in remnant thyroid tissue of berry ligament. The immunohistochemical analysis showed that β-Catenin was diffusely positive in both cytoplasm and nucleus of cancer cells in CMV-PTC. However, normal epithelium of thyroid gland showed membranous staining pattern. Written informed consent is obtained from the patient for publication of the case report and accompanying images.

## 3 Discussion

**Fig. 4:**
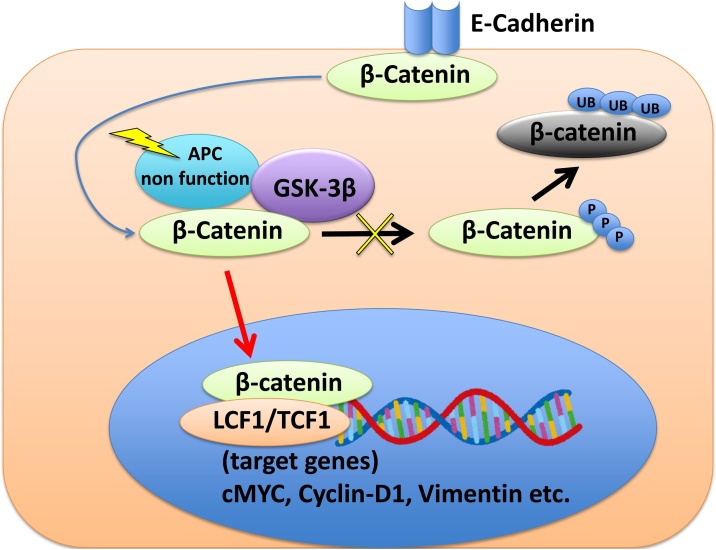
Molecular pathway of CMV-PTC. β-Catenin degrades after phosphorylation. The mutation of APC gene with loss of function leads to overexpress β-Catenin protein because of attenuated phosphorylation of β-Catenin by GSK3β. Over expressed β-Catenin transcript targets genes such as Cyclin-D1, and cMYC, and activates cell proliferation and survival.

The major cause of FAP is the *APC* gene, which is located at the 5q21 locus [9]. Individuals with a germ line *APC* gene mutation have an almost 100% risk of developing colorectal cancer during their lifetime [10,11]. APC and β-Catenin, wnt signaling pathway, also have the most important role in CMV-PTC. The CMV-PTC has the unique pathogenesis associated with *APC* gene mutation, whereas conventional PTC has *BRAF* as well as *RAS* mutation, and *RET/PTC* rearrangement. In CMV-PTC, germline mutations, somatic mutations and loss of heterozygosity of *APC* gene are frequently observed. The mutation of *APC* gene with loss of function leads to overexpress β-Catenin protein because of attenuated phosphorylation of β-Catenin by GSK3β. Overexpressed β-Catenin transcript targets genes such as Cyclin-D1, and cMYC, and activates cell proliferation and survival (Fig. 4). One third of CMV-PTC did not have *APC* gene mutation [12]. Interestingly, the mutation of β-Catenin gene, *CTNNB1*, which accumulates β-Catenin also showed CMV-PTC [13]. This mutation was considered to occur in the non-FAP-associated CMV-PTC.

β-Catenin and E-Cadherin play a crucial role in cell-to-cell adhesion and maintaining epithelial morphology [14,15]. This cadherin/catenin complex also regulates cell motility and believed to function as an invasion suppressor. In CMV-PTC, E-Cadherin was known to be strongly and diffusely membranous positive in morular cells.

Uchino S et al. showed that the multiple lesions of CMV-PTC have different somatic gene mutations with same germ line mutation [12]. This discovery implies that multiple cancers of CMV-PTC will be derived as the *de novo*, not intra-thyroid metastasis. The CMV-PTC have better prognosis than conventional PTC, which have over 90% disease free survival and over 98% over all survival at 10 years after surgery [6]. In the literature 8.5% of CMV-PTC showed recurrence and only 2% patients died of the disease [6]. However, the *de novo* CMV-PTC cancer will occur in the remnant thyroid when total thyroidectomy was not performed in the initial surgery. Recurrent surgery is known as a strong risk factor of postoperative RLN palsy due to adhesion of primary surgery unless using intraoperative neuromonitoring [[16], [17], [18]]. In case of CMP-PTC with FAP, total thyroidectomy is sufficient for the treatment of most cancers. And lymph node dissection could perform when it is necessary.

## 4 Conclusion

We have reported our experience with a rare case of the recurrent surgery for CMV-PTC. The strong expression of β-Catenin is a hallmark staining in CMV-PTC. When CMV-PTC with FAP is suspected preoperatively, the total thyroidectomy should be performed including the berry ligament portion.

## Funding

None.

## Ethical approval

In Japan, ethical committee usually are not required for publishing case report.

## Consent

Written informed consent was obtained from the patient for publication of this case report and accompanying images by both Japanese and English.

## Author contribution

Keisuke Enomoto, Shunji Tamagawa, Naoko Kumashiro, Saori Takeda, Mehmet Gunduz, and Muneki Hotomi have treated surgery and postoperative care of patient.

Kenji Warigaya, and Shin-ichi Murata analysed histologically.

Keisuke Enomoto, Mehmet Gunduz and Muneki Hotomi prepared to manuscript.

Kenji Warigaya, and Shin-ichi Murata make Fig. 3 and legend.

## Registration of research studies

None.

## Guarantor

Muneki Hotomi.

## Provenance and peer review

Not commissioned, externally peer-reviewed.

## Declaration of Competing Interest

None.
